# βB1-Crystallin: Thermodynamic Profiles of Molecular Interactions

**DOI:** 10.1371/journal.pone.0029227

**Published:** 2012-01-06

**Authors:** Monika B. Dolinska, Paul T. Wingfield, Yuri V. Sergeev

**Affiliations:** 1 National Eye Institute, National Institutes of Health, Bethesda, Maryland, United States of America; 2 National Institute of Arthritis and Musculoskeletal and Skin Diseases, National Institutes of Health, Bethesda, Maryland, United States of America; Griffith University, Australia

## Abstract

**Background:**

β-Crystallins are structural proteins maintaining eye lens transparency and opacification. Previous work demonstrated that dimerization of both βA3 and βB2 crystallins (βA3 and βB2) involves endothermic enthalpy of association (∼8 kcal/mol) mediated by hydrophobic interactions.

**Methodology/Principal Findings:**

Thermodynamic profiles of the associations of dimeric βA3 and βB1 and tetrameric βB1/βA3 were measured using sedimentation equilibrium. The homo- and heteromolecular associations of βB1 crystallin are dominated by exothermic enthalpy (−13.3 and −24.5 kcal/mol, respectively).

**Conclusions/Significance:**

Global thermodynamics of βB1 interactions suggest a role in the formation of stable protein complexes in the lens via specific van der Waals contacts, hydrogen bonds and salt bridges whereas those β-crystallins which associate by predominately hydrophobic forces participate in a weaker protein associations.

## Introduction

The transparency and refraction of the mammalian lens are dependent on the molecular associations of the crystalline proteins. These proteins form a βγ-crystallin superfamily those members are similar in structure and contain Greek key motifs. The β-crystallins constitute the major proportion of the lens proteins and seven subtypes have been identified, four of which are acidic (βA1, βA2, βA3, and βA4), and three basic (βB1, βB2, and βB3). Most β-crystallins are monomer-dimer systems [1]–[3] but some, for example, βA2 and βA4, have low intrinsic solubility and exhibit only weak self-associations [4], [5]. The *in vivo* heteromolecular interactions of acidic with basic β-crystallins can circumvent solubility issues [4]–[6]. Under physiological conditions only β-crystallins are known to associate into dimers, tetramers, and higher-order oligomers [3], [7]–[9]. Although the interactions of the β-crystallins have been well studied [4], [10], the detailed molecular mechanisms of most associations remain obscure.

We previously demonstrated that the self-associations of both βA3 and βB2 are mediated by hydrophobic interactions [11]. Here we describe the energetics controlling βB1 dimerization and tetramer formation with βA3. βB1 and βA3 crystallins are major component in the human lens [12], [13] and both recently were found in non-lens tissues including the retina [14]–[16]. We show that the molecular associations of βB1 crystallin are dominated by exothermic enthalpy. This indicates that βB1 plays an important role in the formation of stable protein complexes mediated by specific (stronger) interactions stabilized by van der Waals forces, hydrogen bonds, and salt bridges.

## Results

### Protein molecular weights and associative behavior

βB1 (monomer, 28 kDa) and βA3 (monomer, 25 kDa) elute during size-exclusion chromatography (SEC) with apparent molecular weights of 35 and 42 kDa, respectively, intermediate between that of monomers and dimers (**1**). Sedimentation equilibrium analysis indicated the proteins are reversible monomer-dimer systems [2]. When equimolar amounts of βB1 and βA3 were mixed and incubated for 24 hrs at 20°C, a single symmetrical peak with apparent molecular weight of ∼70 kDa was observed (**Fig. S1**). Sedimentation equilibrium analysis of the mixture indicated a weight-average molecular weight of 95 kDa close to that predicted for a weak heterotetramer (*M*_r_ 106 kDa). The best–fit model for the equilibrium data was a heterodimer - heterotetramer system with a *K_d_* of 8.78 µM (**Table S1**). This result is very similar to that measured for the analogous interaction with murine β-crystallins [17].

### βB1 dimerization energetics

**Figure 1:**
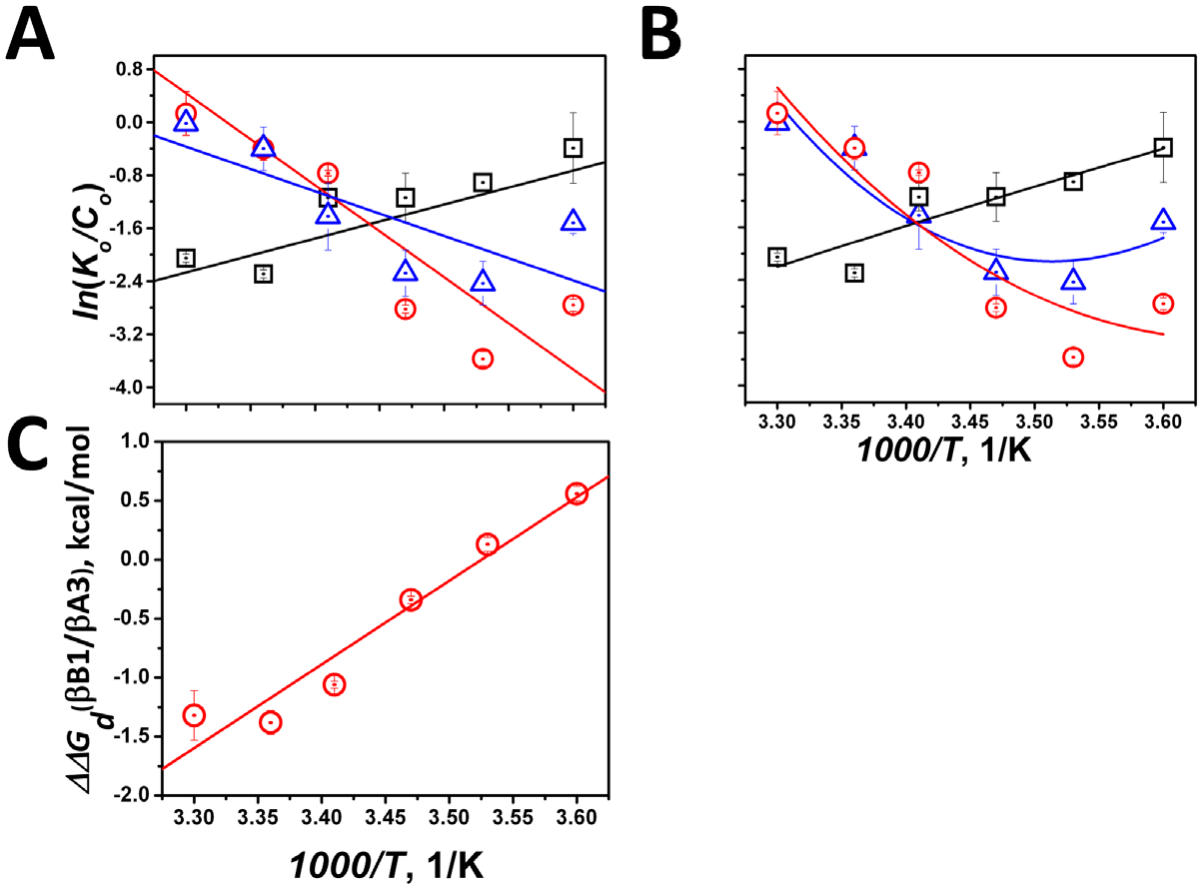
The temperature dependence of changes in the free energies for the dimeric association of βB1 and βA3 and the tetrameric association of βB1/βA3. Asssociation of βB1, βA3 and βB/βA3 are shown by blue open triangles, black open squares and red open circles, respectively. Panels A and B: van't Hoff plots where *ln(K_d_/C_o_)* is plotted as function of the reciprocal of absolute temperature (*1000/T*), *K_d_'s* are the dissociation constants obtained from analytical ultracentrifugation, and *C_o_* is the µM concentration. Panel A: the difference in heat capacity (*ΔC_p_*) is constrained to be 0, resulting in a linear function; Panel B: *ΔC_p_* is not constrained and has a nonzero value. Panel C: temperature dependence of Gibbs free energy gained in formation of βB1/βA3. *ΔΔG_d_ (βB1/βA3)* is defined as a difference between Gibbs free energy changes of tetrameric βB1/βA3 and that of individual components (βB1 and βA3). Concentrations for βB1, βA3, and βB1/βA3 crystallins were each 0.5 mg/ml.

To gain information on the energetics of the βB1 interactions, the temperature dependence of association was determined by sedimentation equilibrium over the range 5–30°C. The equilibrium profiles are shown in **Fig. S2** **Panel A**. βB1 is a reversible monomer- dimer system with the equilibrium position shifting towards monomeric protein at higher temperatures as indicated by increased *K_d_* values (**Table S1**). For example, the dimer fraction of βB1 (total 18 µM) decreased from 81% at 10°C to 50% at 30°C. The calculated free energy *ΔG_a_* values for dimerization also decrease with increase in temperature. This is due to the negative contributions from both enthalpy *ΔH_a_* and entropy *ΔS_a_* (**Table 1**) which were derived from plots (non-linear) of In *K_d_* and *C_o_* versus 1/T (**Fig. 1A, B**).

**Table 1:** Thermodynamic profiles for the associations of homodimeric βB1 and βA3 and tetrameric βB1/βA3.

Crystallin	*ΔC_p_*cal/deg mol	*ΔS_a_*e. u.	*ΔH_a_*kcal/mol	*−TΔS_a_*kcal/mol	*ΔG_a_*kcal/mol
**βB1**	0	−43.2 (±19.3)	−13.3 (±5.6)	12.7 (±5.8)	−0.6
	−2.5 (±0.9)	−69.3 (±15.5)	−21.2 (±4.6)	20.3 (±4.5)	−0.9
**βA3**	0	29.8 (±10.2)	8.0 (±3.0)	−8.7 (±3.0)	−0.7
	−0.8 (±0.6)	27.8 (±8.5)	7.2 (±2.5)	−8.1 (±2.5)	−0.9
**[βB1/βA3]_4_**	0	−81.1 (±21.2)	−24.5 (±6.2)	23.8 (±6.2)	−0.7
	−1.6 (±1.6)	97.9 (±27.4)	−29.6 (±8.1)	28.7 (±8.0)	−0.9

Thermodynamics parameters, enthalpy *ΔH_a_*, and entropy *ΔS_a_* changes were determined using linear (*ΔC_p_ = 0*) and nonlinear (*ΔC_p_≠0*) fitting functions into van't Hoff plots. The Gibbs free energy changes *ΔG_a_* were calculated using formula *ΔG_a_ = ΔH_a_−TΔS_a_*, where *T* is temperature in K; e.u. = 1 cal/(deg mol).

### βA3 dimerization energetics

To be consistent with our previous work with murine βA3 crystallin [11], we analyzed the association behavior of the human protein (sequence identity with murine protein 95%). As expected, human βA3 formed tighter dimers (lower *K_d_*'s) at higher temperatures (**Table S1**) [11]. Thus, in contrast to βB1, the dimer fraction of βA3 increases with temperature (58% at 5°C and 80% at 30°C). There was a linear dependence of the ln*K_0_/C_0_* on *1000/T* using the change in heat capacity *ΔC_p_* zero or non zero values. (**Table S1**, and **Fig. 1A, B**). Hence, the self-association of human βA3 is characterized by positive enthalpy *ΔH_a_* and entropy *ΔS_a_* at zero and nonzero *ΔC_p_* (**Table 1**), which is similar to our previously published analysis of the murine protein [2].

### βB1/βA3 tetramerization energetics

The βB1/βA3 complex is best modeled as a reversible heterodimer-heterotetramer system over the temperature range studied (5–30°C) with a tendency to form weaker tetramers at higher temperature (**Fig. S1, Fig. S2 Panel B,** and **Table S1**). The relationship between the logarithm of the *K_d_* and the reciprocal of the absolute temperature demonstrated by the van't Hoff plot is not linear (**Fig. 1A, B**). When *ΔΔG_a_* is plotted against the reciprocal of the absolute temperature (**Fig. 1C**) *ΔΔG_a_* values negatively increase with increasing temperature. Tetramer formation is, therefore, associated with exothermic enthalpy *ΔH_a_* and entropy *ΔS_a_* (**Table 1**) and, analogous to βB1 dimerization, *ΔG_a_* decreases with increasing temperature (**Fig. 1C**).

## Discussion

We have previously shown that both murine βA3 and βB2 are monomer – dimer systems with a tendency to form tighter dimers at higher temperatures [11]. Moreover, the self-association of these crystallins, characterized by positive enthalpy and entropy changes, is entropically driven and mediated by hydrophobic interactions. These endothermic associations (*ΔH*>0) are dominated by hydrophobic effects entropically driven by water. Here we have shown a similar energetic profile for human βA3 (**Table S1**) indicating that nonpolar regions of the protein, previously accessible to solvent in the isolated subunits, become buried upon dimer formation [18].

The self-association of βB1 energetically differs from that of βA3 and βB2 in that its dimers are destabilized at higher temperatures. The thermodynamic profile (**Table 1**) indicates that both the dimerization of βB1 and formation of the βB1/βA3 complex are exothermic processes (*ΔH*<0). With tetrameric βB1/βA3 formation, decreasing negative values of *ΔG* confirm that the complex is less stable at higher temperatures. Large exothermic enthalpy change *ΔH_a_* = −29.6±8.1 kcal/mol and negative entropy *ΔS_a_* = −97.9±27.4 e.u. are accompanied with a negative heat capacity change *ΔC_p_* = −1.6±1 cal/deg mol. Thus, the profile suggests that tetramer formation is controlled by enthalpy and interactions between the subunits are mediated by van der Waals interactions, hydrogen bonds, and salt bridges [18].

**Figure 2:**
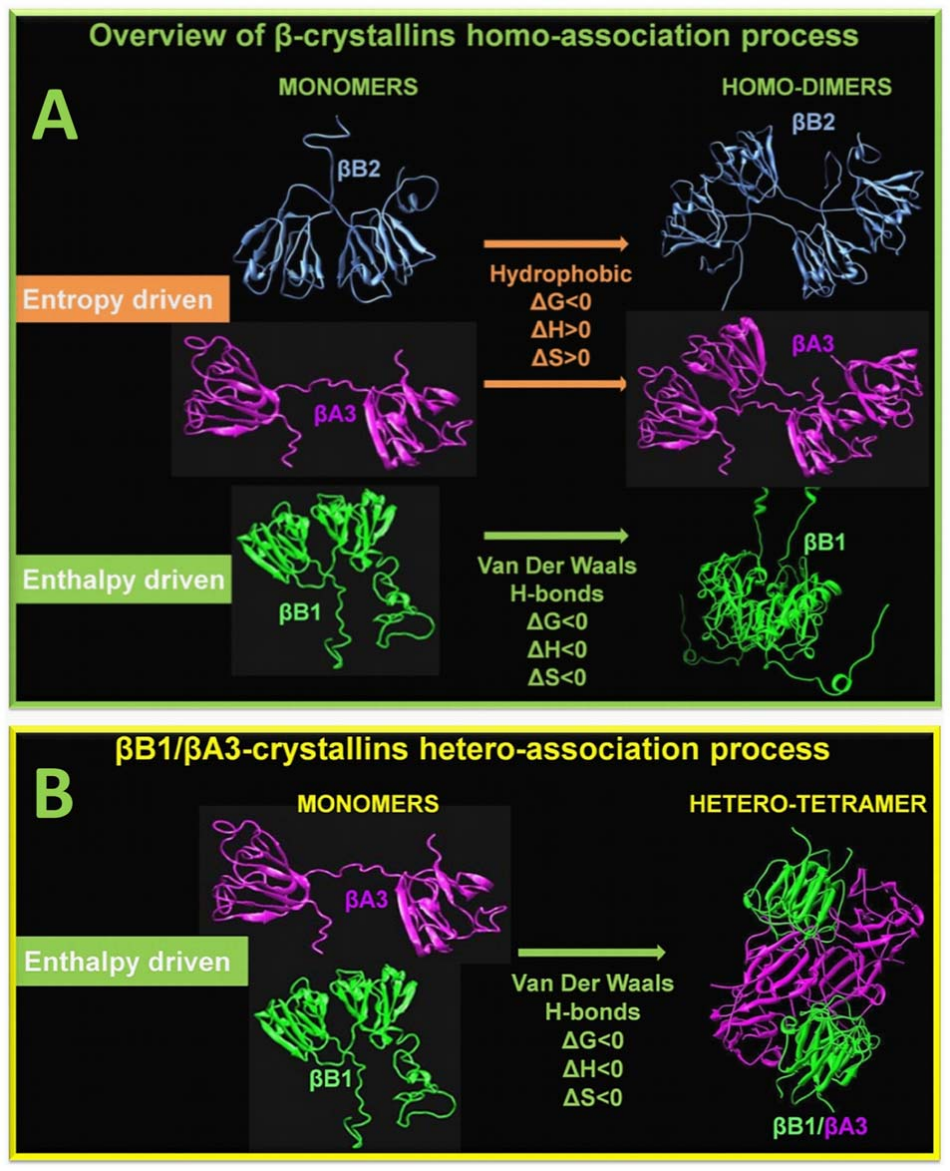
Overview of the homo- and heteromolecular associations of β-crystallins. Top panel: βA3, βB1, and βB2 self-associate in a reversible manner to form dimers. The homo-associations of βA3 and βB2 exhibit endothermic enthalpy and are driven by entropy as a result of hydrophobic interactions between protein molecules. In contrast, the self-association of βB1 is driven by exothermic enthalpy due to van der Waals interactions and hydrogen bonds at the dimer interface. Bottom panel: The βB1/βA3 complex is likely formed by the association of hetero-dimers but we cannot rule out that it is formed from homodimers. Similar to that of βB1 alone, the formation of the tetramer is driven by exothermic enthalpy. Structures of βB1 and βB2 were obtained from the protein database RCSB (files: 1 oki and 1 blb, respectively). Closed and open structures of βA3 were modeled as described earlier [1]. From our results we cannot say which monomer conformation exists within the hetero-tetramer. However, the majority of known crystal structures of β-crystallins (3 of 4) are of the closed monomer type suggesting this is the most stable conformation. Therefore, the structure of the hypothetical tetrameric βB1/βA3 complex was generated using the crystal packing of βB1 crystallin as a template (PDB file: 1 oki).

A summary overview of the homo- and hetero-associations of β-crystallins is presented in **Fig. 2**. βB1 mediates protein interactions using van der Waals contacts and hydrogen bonds which suggest that contact involve complementary shapes of protein surfaces with a higher biological specificity [19]. In contrast the formation of dimeric βA3 and βB2, are driven by hydrophobic forces which are usually less specific. In these associations, hydrophobic residues at the surface interfaces become excluded from direct contact with surrounding water molecules.

Currently we cannot completely rule out the possibility that heterotetramers are formed by the association of homodimers rather than heterodimers but in either case it does not affect our analyses and conclusions. However; the precise mechanism of βB1/βA3 association appears to involve the association of heterodimers [2]. The kinetics, equilibrium position and balance of so-called “close” and “open” conformational isomers could be affected by interactions of core domains or with the N-terminal extensions of βA3 and βB1 [1], [17]. The dimerization of βB1 may ‘induce’ a conformational shift which favors interaction with βA3. Such a model would explain why both, homo- and hetero-association of βB1 are driven by enthalpy.

Previous dynamic light scattering analysis demonstrated that in fetal calf lenses soluble crystallins form a broad distribution of protein complexes with sizes of 8–14 nm with β- and γ-crystallins at the leading edge of this distribution [20]. Proteomic analysis has demonstrated that large molecular complexes are often built around a stable core of proteins, which are expanded thorough the attachment of weakly bound exchangeable peripheral proteins often stabilized by dynamic transient interactions [21], [22]. These exchangeable components could be for example, so-called ‘weak’ dimers which have relatively high *K_d_*'s [23]. The protein interfaces in ‘weak’ dimers are loosely packed and more hydrophobic than in average protein transient complexes.

βB2 and βA3 crystallins could have a propensity to be components of a peripheral protein network. The less specific and more transient nature of their interactions would give these crystallins more flexibility for binding. On the other hand, the more specific and stronger exothermic interactions involving βB1 make this crystallin more suitable for formation of the stable core of the lens proteins.

Although we have described the interactions of the crystallins as being mediated by either the weaker hydrophobic or the more specific van der Waals interactions, both may occur but on average one dominates energetically. It is known, for example, from antibody – antigen interactions, that initial contacts may involve hydrophobic interactions via interface aromatic residues followed by more specific and tighter H- bonding and salt bridges [18], [24].

In conclusion, the global thermodynamics of βB1 interactions indicate that they contribute in more stable protein complexes in the lens via specific van der Waals contacts, hydrogen bonds and salt bridges whereas those β-crystallins which associate by predominately hydrophobic forces are more likely to participate in a weaker protein associations.

## Materials and Methods

### Expression, purification and association of βB1- and βA3-crystallins

Wild type recombinant murine βB1 and human βA3 were expressed as soluble proteins in *E.coli* and purified as previously described by ion-exchange and size-exclusion chromatographies [2], [17]. Murine βB1 was used which has a 95% sequence similarity (>80% sequence identity) with the human protein and is, therefore, a reasonable surrogate. The purified proteins were dialyzed overnight against Buffer A (50 mM Tris-HCl, 1 mM EDTA, 0.15 M NaCl, 1 mM TCEP, at pH 7.5) at 4*°C*. Protein concentrations were estimated from A_280/260_ (Beckman Coulter DU650, CA) and adjusted to 0.5 mg/ml. For the formation of complexes between βB1 and βA3, an equimolar mixture (∼20 µM each) was incubated at room temperature for 24 h. Aliquots (250 µl) were loaded on an analytical grade Superdex 75 HR10/30 column, precalibrated with standards (bovine serum albumin, 67 kDa, ovalbumin, 43 kDa, chymotrypsinogen, 25 kDa, and ribonuclease A, 13.7 kDa; Sigma, MO). Samples were eluted at a flow rate of 0.5 ml/min and 0.5 ml fractions were collected.

### Analytical Ultracentrifugation

A Beckman Optima XL-I analytical ultracentrifuge with absorption optics, an An-60 Ti rotor, and standard double-sector centerpiece cells were used for sedimentation equilibrium experiments. All analyses were performed using duplicate protein samples. Data were collected after 16 hours at 18,500 rpm at 20°C. The baselines were established by overspeeding at 45,000 rpm for another 4 hours. Equilibrium profiles were analyzed by standard Optima XL-I Origin-based data analysis software. Solvent density was estimated as previously described [25]. Monomeric molecular weights *M_r_* and molar extinction coefficients were used for calculation of dissociation constants *K_d_*. The *M_r_* and *K_d_* were measured in duplicate and averaged. Equilibrium data was collected with 5*°C* temperature increments for the ranges: 5–25°C and 15–30°C.

### Energetics of monomer-dimer and dimer-tetramer equilibrium

The temperature dependence of association was examined for homodimer and hetero-tetramer associations between 5–30*°C*, using a previously described [11] equation:
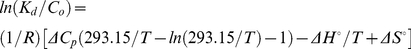
(1)where *K_d_* is the dissociation constant, measured by AUC; *C_o_* is the molar concentration of protein (µM); *R* is universal gas constant; *T* is temperature (K); and *ΔC_p_*, *ΔH°*, and *ΔS°* are changes in protein heat capacity, enthalpy and entropy, respectively. The experimental data were fitted in two ways: first; where *ΔC_p_* was constrained to be zero and second; where *ΔC_p_* was nonzero. The effect of protein concentration was excluded from the analysis by normalization to the protein molar concentration *C_o_* (See formula 1).

## Supporting Information

Figure S1 **Size-exclusion chromatography profiles obtained for individual proteins and the βB1/βA3 complex.** The chromatographic profile obtained immediately after mixing of equimolar amounts of βB1 and βA3 is shown in green and following 24 hours of incubation, by the red line. The elution positions of molecular weight standards are shown at the top of the figure.
(TIF)

Figure S2 **Sedimentation equilibrium profiles of βB1 and βB1/βA3 complex at various temperatures.** In the main panels, open circles show the protein concentration profile represented by the UV absorbance gradients in the centrifuge cell at 280 nm. The solid lines indicate the calculated fits for the βB1monomer-dimer (Panel A) or heterodimer – heterotetramer βB1/βA3 complex (Panel B) associations. Residuals in the smaller upper panels show the difference in the fitted and experimental values as a function of radial position. In many of the profiles, the residuals at the bottoms of the cells reveal systematic patterns indicative of aggregating protein; this data was not included in the analyses.
(TIF)

Table S1 **Dissociation constants (** ***K_d_*** **) and changes in Gibbs free energy of association obtained at different temperatures for the formations of dimeric βB1, and βA3, and tetrameric βB1/βA3 Association free energy change** ***ΔG_a_*** **estimated as** ***ΔG_a_ = −ΔG_d_*** **, where the dissociation free energy change is** ***ΔG_d_ = −RT ln(K_d_/C_o_); K_d_*** **is the dissociation constant in µM and** ***C_o_*** **is the protein sample concentration in µM; standard errors were calculated from 2–3 times repeated data and are shown in parentheses.**
(DOCX)
