# Supplementary figures and images for: βB1-Crystallin: Thermodynamic Profiles of Molecular Interactions
Source: PLoS One. 2012 Jan 6;7(1):e29227. doi: 10.1371/journal.pone.0029227 (PMC3253074; doi:10.1371/journal.pone.0029227)

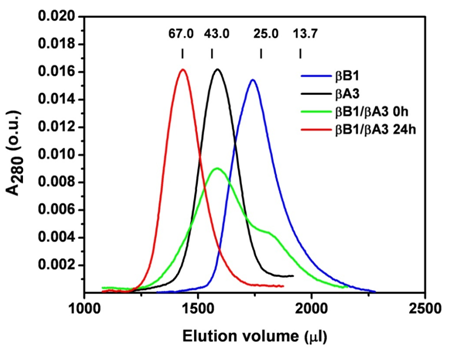

Supplement: Figure S1 — Size-exclusion chromatography profiles obtained for individual proteins and the βB1/βA3 complex. The chromatographic profile obtained immediately after mixing of equimolar amounts of βB1 and βA3 is shown in green and following 24 hours of incubation, by the red line. The elution positions of molecular weight standards are shown at the top of the figure. (TIF) [file pone.0029227.s001.tif]

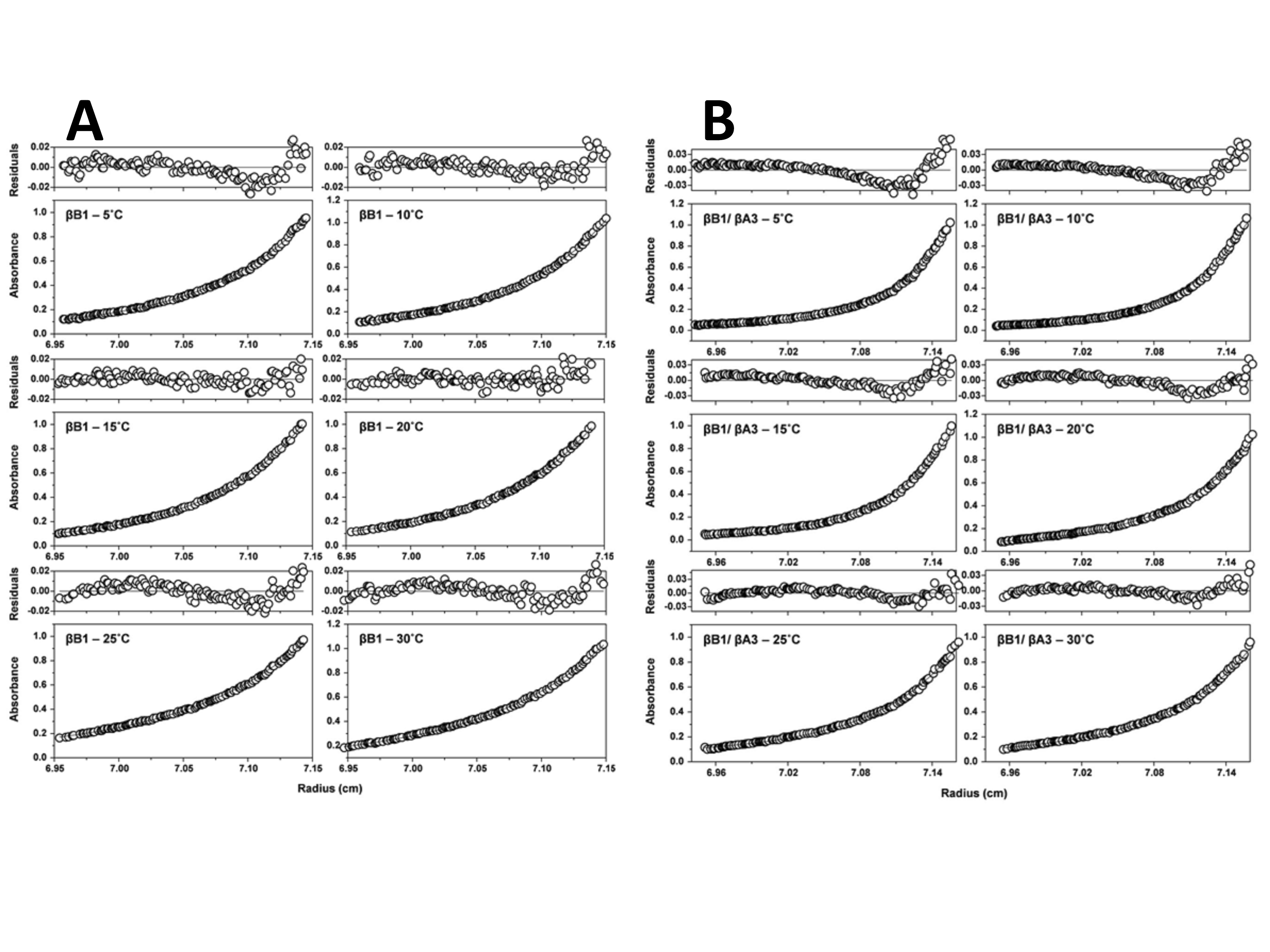

Supplement: Figure S2 — Sedimentation equilibrium profiles of βB1 and βB1/βA3 complex at various temperatures. In the main panels, open circles show the protein concentration profile represented by the UV absorbance gradients in the centrifuge cell at 280 nm. The solid lines indicate the calculated fits for the βB1monomer-dimer (Panel A) or heterodimer – heterotetramer βB1/βA3 complex (Panel B) associations. Residuals in the smaller upper panels show the difference in the fitted and experimental values as a function of radial position. In many of the profiles, the residuals at the bottoms of the cells reveal systematic patterns indicative of aggregating protein; this data was not included in the analyses. (TIF) [file pone.0029227.s002.tif]
