# Supplementary material for: βB1-Crystallin: Thermodynamic Profiles of Molecular Interactions
Source: PLoS One. 2012 Jan 6;7(1):e29227. doi: 10.1371/journal.pone.0029227 (PMC3253074; doi:10.1371/journal.pone.0029227)
Supplement: Table S1 — Dissociation constants ( Kd ) and changes in Gibbs free energy of association obtained at different temperatures for the formations of dimeric βB1, and βA3, and tetrameric βB1/βA3 Association free energy change ΔGa estimated as ΔGa = −ΔGd , where the dissociation free energy change is ΔGd = −RT ln(Kd/Co); Kd is the dissociation constant in µM and Co is the protein sample concentration in µM; standard errors were calculated from 2–3 times repeated data and are shown in parentheses. (DOCX) [file pone.0029227.s003.docx]

**Table S1.**

| ***T*,˚C** | ***K_d_*, µM** | | | ***ΔG_a_*, kcal/mol** | | |
| --- | --- | --- | --- | --- | --- | --- |
|  | **βB1** | **βA3** | **βB1/βA3** | **βB1** | **βA3** | **βB1/βA3** |
| **5** | 3.99 (± 0.64) | 11.80 (± 6.05) | 1.21 (± 0.11) | -0.91 (± 0.10) | -0.18 (± 0.09) | -1.65 (± 0.06) |
| **10** | 1.64 (± 0.54) | 6.47 (± 0.76) | 0.54 (± 0.05) | -1.46 (± 0.21) | -0.55 (± 0.06) | -2.14 (± 0.06) |
| **15** | 1.91 (± 0.67) | 5.32 (± 1.92) | 1.14 (±0.06) | -1.37 (± 0.21) | -0.66 (± 0.24) | -1.69 (± 0.03) |
| **20** | 4.71 (± 2.00) | 5.21 (± 1.07) | 8.78 (± 0.4) | -0.85 (± 0.31) | -0.67 (± 0.14) | -0.46 (± 0.03) |
| **25** | 12.58 (± 4.47) | 1.62 (± 0.09) | 12.80 (± 2.12) | -0.24 (± 0.20) | -1.38 (± 0.08) | -0.24 (± 0.10) |
| **30** | 17.58 | 2.07 (± 0.14) | 22.10 (± 7.21) | -0.01 | -1.23 (± 0.08) | ­0.08 (± 0.21) |
